# The immunogenic reaction and bone defect repair function of ε-poly-L-lysine (EPL)-coated nanoscale PCL/HA scaffold in rabbit calvarial bone defect

**DOI:** 10.1007/s10856-021-06533-7

**Published:** 2021-06-07

**Authors:** Bin Tian, Na Wang, Qingsong Jiang, Lijiao Tian, Lei Hu, Zhenting Zhang

**Affiliations:** 1 Department of Prosthodontics, Beijing Stomatological Hospital, School of Stomatology, Capital Medical University, Beijing, China; 2 Liangxiang Hospital of Beijing Fangshan District, Beijing, China; 3 Beijing Key Laboratory of Tooth Regeneration and Function Reconstruction, Capital Medical University School of Stomatology, Beijing, 100050 China

**Keywords:** ε-poly-L-lysine (EPL), Nanoscale scaffold, Bone tissue engineering, Immune reaction, Calvarial defect

## Abstract

Tissue engineering is a promising strategy for bone tissue defect reconstruction. Immunogenic reaction, which was induced by scaffolds degradation or contaminating microorganism, influence cellular activity, compromise the efficiency of tissue engineering, or eventually lead to the failure of regeneration. Inhibiting excessive immune response through modulating scaffold is critical important to promote tissue regeneration. Our previous study showed that ε-poly-L-lysine (EPL)-coated nanoscale polycaprolactone/hydroxyapatite (EPL/PCL/HA) composite scaffold has enhanced antibacterial and osteogenic properties in vitro. However, the bone defect repair function and immunogenic reaction of EPL/PCL/HA scaffolds in vivo remains unclear. In the present study, three nanoscale scaffolds (EPL/PCL/HA, PCL and PCL/HA) were transplanted into rabbit paraspinal muscle pouches, and T helper type 1 (Th1), T helper type 2 (Th2), T helper type 17 (Th17), and macrophage infiltration were analyzed after 1 week and 2 weeks to detect their immunogenic reaction. Then, the different scaffolds were transplanted into rabbit calvarial bone defect to compare the bone defect repair capacities. The results showed that EPL/PCL/HA composite scaffolds decreased pro-inflammatory Th1, Th17, and type I macrophage infiltration from 1 to 2 weeks, and increased anti-inflammatory Th2 infiltration into the regenerated area at 2 weeks in vivo, when compared to PCL and PCL/HA. In addition, EPL/PCL/HA showed an enhanced bone repair capacity compared to PCL and PCL/HA when transplanted into rabbit calvarial bone defects at both 4 and 8 weeks. Hence, our results suggest that EPL could regulate the immunogenic reaction and promote bone defect repair function of PCL/HA, which is a promising agent for tissue engineering scaffold modulation.

## Introduction

Bone tissue defects are frequently occurring craniomaxillofacial issues that arise from multiple sources including tumors, trauma, congenital malformations, and senile osteoporosis [1]. Tissue engineering is a promising strategy for bone tissue defect reconstruction. Past decades, in order to improve the tissue repair efficacy, numerous researches have focus on the scaffold properties, degradation, and clearance [2, 3]. Despite the great achievement have been made, challenges and obstacles still exist, which limit the tissue repair efficacy and clinical application [4].

The cross talk between scaffold and tissue plays a central role in tissue engineering [5, 6]. Thus, clarifying and regulating the biology interaction between scaffold and tissue are critically important to the improvement of the tissue repair efficacy. Traditionally, the process of bone tissue engineering includes mainly osteoclasts, osteoblasts, and mesenchymal stem cell. Recently, researchers have found that tissue engineering process can trigger innate and adaptive immune reactions. Various immune cells can infiltrate the transplanted area during tissue engineering [7]. Immune cells, including pro-inflammatory cells (T helper type 1 (Th1), T helper type 17 (Th17), type I macrophages) and anti-inflammatory cells (T helper type 2 (Th2), regulatory T cells, type II macrophages), are essential for tissue formation, remodeling, and repair, by releasing regulatory molecules, which can affect osteoclastogenesis and osteogenesis. Abnormal immune reaction toward scaffold induced by scaffold degradation or a contaminating microorganism may lead to chronic inflammation, swelling, pain, fever, fibrous formation, and transplantation failure [8, 9], which would increase difficulty in later treatment. Hence, minimizing the improper immune reaction triggered by scaffolds must be carefully explored.

The host immune response induced by scaffolds due to their physical, chemical, and biological properties [10]. Materials such as polyethylene glycol, Poly (caprolactone) (PCL), or poly (lactic-co-glycolic acid) display low cellular attachment capacity, and can influence inflammatory response [11, 12]. In order to promote tissue regeneration, researchers modulated scaffold immune response by encapsulation of immune regulation molecules, or anti-inflammatory drugs [11]. However, as the immune reaction is a natural process during tissue engineering, the excessive anti-inflammatory or pro-inflammatory could impair the tissue repair process. Thus, a novel effective and gentle immune regulator should be well-established.

E-poly-L-lysine (EPL) is a cationic polypeptide naturally produced by *Streptomyces* or *Kitasatospora* [13]. It is easily soluble in water and can be broken down into lysine or amine, amide, hydroxyl, or carboxyl groups that can promote cell adhesion and growth [14]. Importantly, EPL have antimicrobial activity against a wide spectra of microorganisms and it is also biodegradable and nontoxic to humans [15, 16]. Thus, as a natural antimicrobial, EPL is widely used in the fields of food preservation or medicine, for its strong antibacterial activity and low toxicity. Recently, more and more function of EPL except antibacterial have been found, like glucose sensors, antioxidant, and antitumor activity [17, 18]. Besides, evidences have showed that EPL can improve drug absorption, which is a superior biomaterial for drug delivery [19–22]. It also has showed as a promising drug or gene carriers for bone marrow cells or tissue healing [23, 24], and may be used for bone infection treatments as well as regenerative bone grafts [25]. Importantly, EPL can regulate Th1-mediated immune response combined with other scaffold, like hydrogels or bioglass [26]. But the exact function of EPL on bone regeneration and immune response of scaffold was largely unknown.

Our previous study has successfully constructed 3D EPL-coated nanoscale PCL/hydroxyapatite (EPL/PCL/HA) composite scaffold by Fused Deposition Modeling (FDM)-3D printing technology. Our data showed that EPL-coated 3D EPL/PCL/HA scaffold has antibacterial properties and it also can promote the osteogenic differentiation of osteoblast [27]. However, the bone defect repair functions of EPL/PCL/HA in vivo remain unclear. This study explored bone defect repair function of an EPL/PCL/HA composite scaffold in vivo using a rabbit calvarial bone defect model. The immunogenic reaction of EPL/PCL/HA was also detected by immunohistochemistry (IHC) staining for Th1, Th2, Th17, and macrophage.

## Materials and methods

### Animals

Sixty male New Zealand white rabbits (20–22 weeks old, 2.8 ± 0.3 kg) were obtained from the School of Stomatology, Capital Medical University, Beijing, China. The animals were kept under conventional conditions (at room temperature with 12 h of light and 12 h of dark) with free access to water and standard rabbit food. Five weeks after surgery, rats were sacrificed by utilizing an overdose of chloral hydrate (200-mg/kg body weight). Animal death was verified by observation of cardiac arrest and pupil enlargement for 1 min. This study was reviewed and approved by the animal care and use committee of Capital Medical University (KQYY-201803-002).

### Preparation of PCL, PCL/HA, and EPL/ PCL/HA composite scaffolds

Since the bone regeneration scaffold has a porosity of 50–90% and a pore size of 300–600 μm, our previous study optimized the composition of EPL/PCL/HA with a PCL:HA ratio of 7:3, pore size of 450 μm and porosity of 50% [27]. The scaffolds were prepared as follows. Briefly, we obtained nanoscale HA (Kunshan Overseas Chinese New Material Co., Ltd, China) and PCL (Shenzhen Esun Industrial Co., Ltd, China), respectively. The PCL and HA particles were mixed with the mass ratio as 7:3 in high temperature. Then PCL and PCL/HA scaffold were constructed with the FDM system (Jiangyin Recongene Biomedical Technologies, Ltd Inc., Jiangyin, China). The mini-extruder system outputs the PCL or PCL/HA in a molten form through the deposition nozzle, with the nozzle diameter of 300 μm. The melting temperature of upper equipment was set at 75 °C, and the melting temperature of nozzle tip was maintained at 110 °C during the fabrication process. The scaffolds were fabricated into a 3D model with a pore size of 300 μm and pitch of 450 μm. After preparing the scaffolds, it must be aseptically packaged and sterilized with cobalt-60. Our previous study has showed that 5-mg/ml EPL-coated PCL/HA would witness a stable release curve over 3 days [27]. Thus, for EPL/PCL/HA scaffolds preparation, PCL/HA scaffolds were immersed in 5-mg/ml EPL (Zhengzhou Bainafo Bioengineering Co., Ltd, China, dissolved in sterile deionized water). After 24 h, the solution was aspirated. EPL/PCL/HA scaffolds were dried in a sterile drying cabinet and sealed in sterile storage at 4 °C and used within 1 week.

### Scaffold implantation into rabbit paraspinal muscle pouches

All surgical procedures were performed under a strict aseptic protocol. Forty rabbits were anesthetized with a mixture of Zoletil 10 mg/kg (Zoletil 20, Virbac, France) and Sumianxin II 0.2-ml/kg injected intramuscularly to the hind leg. The backs of the rabbits were shaved and disinfected with 0.5% iodophor solution (Beijing Sihuan Sanitary Pharmacy Factory Co., Ltd., Beijing, China), and an incision was made to expose the paraspinal muscle. After three incisions were made into the muscle fascia on both sides of the spine, three muscle pouches were created by blunt separation. One of each scaffold (PCL, PCL/HA, and EPL /PCL/HA) was implanted into each muscle pouch of each rabbit. After the scaffolds were placed, the wounds were closed in layers using silk sutures. Tissues from scaffolds were collected at 1 and 2 weeks, followed by IHC analysis, collected at 4 and 8 weeks for hematoxylin and eosin (H&E) staining (*n* = 10).

### Scaffold implantation in rabbit skull bone defects of critical size

Using the same anesthetic method as the previous experiment, twenty rabbits were shaved from the eyes to the occipital eminence and between the ears. Their skin was disinfected as in the previous experiment. A midline sagittal incision was made and the skin and periosteum were carefully raised so that the cranium was revealed. Four critical size defects were prepared using drills motivated by a surgical micro motor at 800 rpm (Bien air, Switzerland) and under abundant cooled physiological saline; two to each side of the sagittal suture, keeping away from the frontal or occipital sutures [28]. The edge of the bone defect was 2 mm from the middle slit and marked with a 1-mm diameter metal nail to facilitate the determination of the center point of the bone defect during the subsequent measurement and analysis. The bone discs were carefully sectioned and removed with a periosteal stripper. Four critical size defects of each rabbit were randomly divided into four treatment groups (blank (no scaffold) and PCL, PCL/HA, and EPL/PCL/HA scaffolds). The periosteum and skin were sutured, respectively. An intramuscular injection of benzathine ampicillin (50 mg/kg) was given in the immediate postoperative period and again within 48 h after the surgery. Tissues were collected at 4 and 8 weeks, respectively.

### Fluorescent labeling

Animals were injected with 10-mg/ml (10-mg/kg body weight) Calcein AM (*λ*ex 495 nm; *λ*em 515 nm, Sigma) at 13 and 14 days before the animals were euthanized and 10-mg/ml Alizarin Red S (*λ*max 567 nm, Sigma) fluorescent injection was intravenously injected 3 and 4 days before the animals were euthanized. The fluorescent label adhered to the new bone. Under a fluorescence microscope, the calcein fluorescent agent appeared bright green and the alizarin red fluorescent agent appeared red.

### Fluorescence and toluidine blue staining analysis

Ten animals each from skull bone defect transplant groups were euthanized at 4 and 8 weeks after surgery. The animals were deeply anesthetized and killed with overdose injection of pentobarbital. The tissues derived from PCL, PCL/HA, and EPL/PCL/HA groups were removed as a whole and immersed in 4% buffered formaldehyde. After completely fixing the specimens, samples were dehydrated with a graded series of ethanol concentrations before being embedded in resin and sliced into 20-μm-thick sections for observation by fluorescence microscopy (Olympus BX/TF, U-LH100HG, Tokyo, Japan). Newly formed mineralized bone in the defect regions was observed using Image J software based on the gray values. In addition, sections were stained with toluidine blue and the amount of newly formed mineralized bone was calculated using Image J image-analysis software (National Institutes of Health, USA). Newly mineralized bone (%) = newly formed mineralized bone area/total defect area.

### Histomorphometric analysis

Tissues were decalcified with 10% ethylenediamine tetraacetic acid (pH 8.0), embedded in paraffin, and sliced into 5-μm sections followed by H&E staining and Masson’s trichrome staining according to the manufacturer’s protocols.

IHC analysis was used to investigate the immune response of the scaffolds after in vivo transplantation. T-bet, GATA3, ROR gamma T, CD68, CCR7, and CD206 levels were analyzed as markers for Th1, Th2, Th17, pan-macrophages, type I macrophages, and type II macrophages, respectively. Tissues derived from the PCL, PCL/HA, and PCL/HA/EPL groups were harvested at 4 and 8 weeks, fixed with 10% formalin, embedded in paraffin, and sliced into 5-μm sections. The sections were deparaffinized, underwent antigen retrieval in boiled sodium citrate buffer solution (pH 6.0), then cooled at room temperature for 1 h. Sections were blocked in blocking buffer and incubated with primary antibodies: anti-T-bet (1:1000, Thermo Fisher), anti-GATA3 (1:1000, LSBio), anti-ROR gamma/RORC/NR1F3 (1:1000, Novus), anti-CD68 (1:1000, CST), anti-CCR7 (1:10000, Abcam), and anti-CD206 (1:1000, CST). Antibodies were diluted with dilution buffer and incubated at 4 °C overnight. Samples were then washed in Tris Buffered Saline with Tween (TBST) for 5 min and anti-rabbit IgG-HRP (1:1000, Santa Cruz, Dallas, Texas, USA) was used to detect primary antibody signals after 1 h incubation at room temperature. 3,3′-diaminobenzidine (DAB) staining was performed using a SignalStain^®^ DAB Substrate Kit (Cell Signaling, Danvers, MA, USA). The results were captured using a Leica DM 4000 microscope (Leica, Germany).

All samples were stained in triplicate. Triplicate sections were separately evaluated by two blind observers with a digital microscope (Olympus BX/TF, Tokyo, Japan) and a digital camera (Olympus, Tokyo, Japan) at ×20 magnification. The sections were evaluated using Image J software (National Institutes of Health, USA). The following histomorphometric parameter was determined: new bone area ratio (%) = (newly formed bone area, mm^2^)/(total area, mm^2^) × 100.

### Statistical analysis

All data were expressed as the mean ± standard deviation. A one-way ANOVA was performed for multiple comparisons among all groups (Duncan’s multiple range test) using SPSS 20.0 software (SPSS Inc., Chicago, IL, USA). Differences were considered statistically significant when *p* < 0.05.

## Results

### EPL/PCL/HA promotes tissue regeneration in the muscle transplant model

**Fig. 1:**
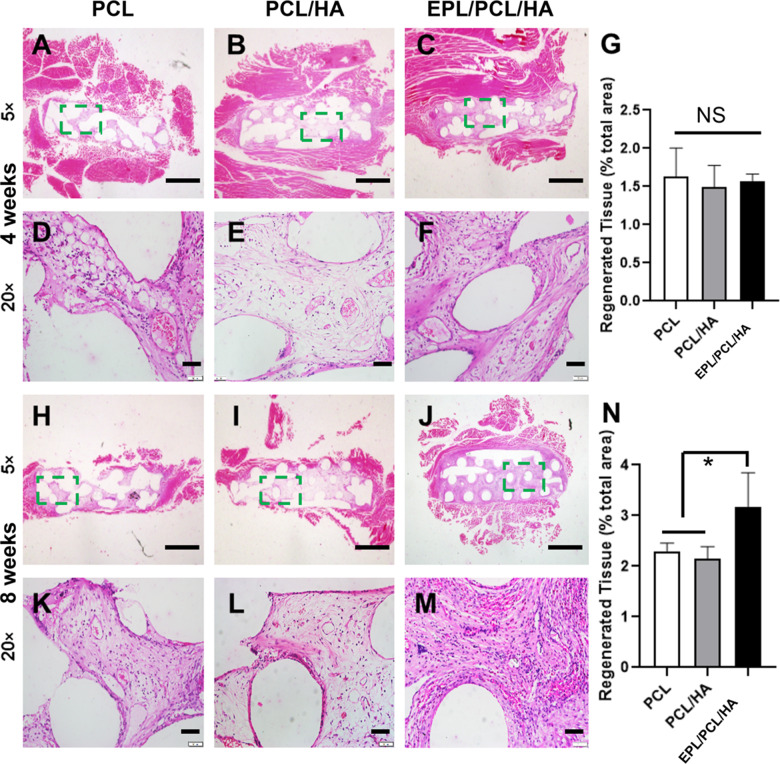
Hematoxylin–eosin staining of tissue samples treated with PCL, PCL/HA, and EPL/PCL/HA composite scaffolds. **A–F** and **H–M** present the histological findings at 4 and 8 weeks, respectively. **G, N** Regenerated tissue (% total area) is reflected by the ratio of new tissue area to the total scaffold area (*n* = 10, *p < 0.05)

After the PCL, PCL/HA, and EPL/PCL/HA scaffolds implanted into the rabbit muscle, all postoperative healing was generally uneventful. No complications (swelling, redness, infection, dehiscence, or exposure) were observed throughout the healing period. At 4 weeks, many cells had infiltrated the scaffold margin and only a small amount of new tissue was observed inside all three scaffold types. The amount of new tissue did not differ significantly between groups (Fig. 1A–G). At 8 weeks, the cells and tissues were significantly increased compared to those at 4 weeks in all three groups. The boundaries between the materials and surrounding tissues gradually blurred, with new tissues growing inside the scaffold while the scaffold structure collapsed. The regenerated tissue area in the EPL/PCL/HA scaffold group was about 50% higher than that in the PCL and PCL/HA groups (Fig. 1H–N).

### EPL/PCL/HA regulates Th1, Th2, Th17, and macrophage I/II levels in regenerated tissues

**Fig. 2:**
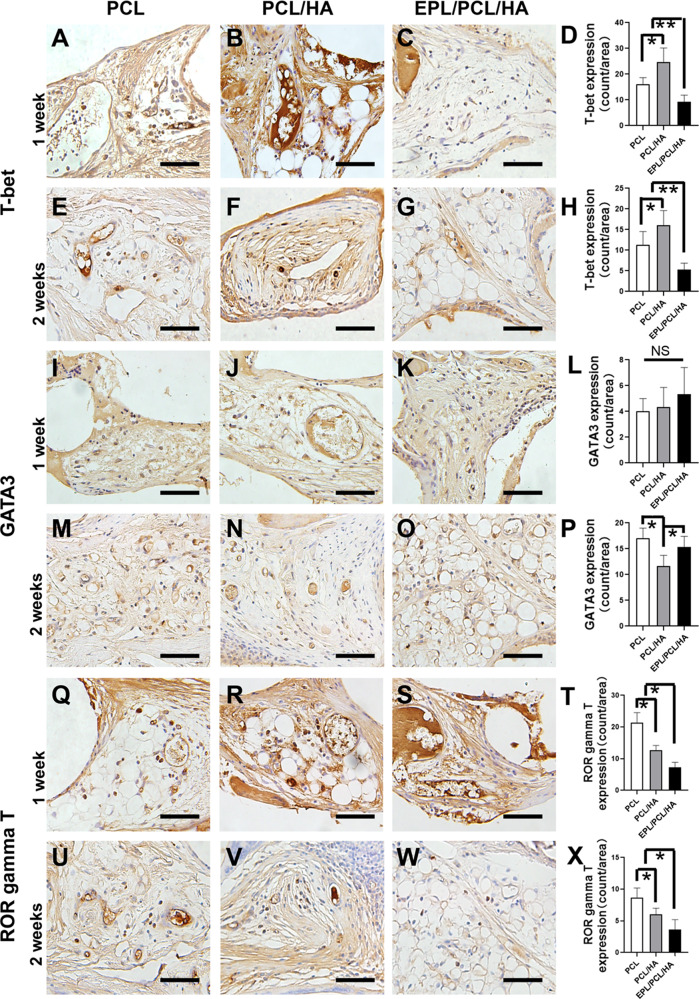
Representative images of immunohistochemical staining for T-bet, GATA3, and ROR gamma T in PCL, PCL/HA, and EPL/PCL/HA groups. Th1, Th2, and Th17 cells were recognized as positive markers for T-bet (**A–C, E–G**), GATA3 (**I–K, M–O**), or ROR gamma T (**Q–S, U–W**). T-bet, GATA3, and ROR gamma T levels were statistically analyzed (**D, H, L, P, T, X**). All data are given as the mean ± SEM (*n* = 10). *p < 0.05, **p < 0.01

**Fig. 3:**
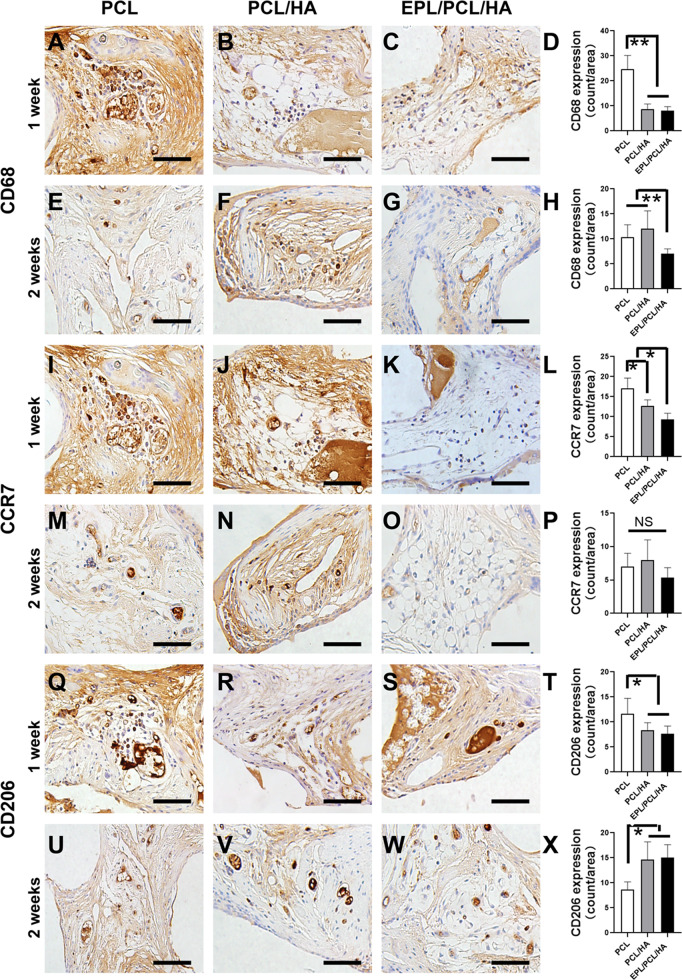
Effects of PCL, PCL/HA, and EPL/PCL/HA scaffolds on macrophage polarization measured by immunohistochemistry in regenerated tissues. Representative images of immunohistochemical staining for CD68, CCR7, and CD206 in all three groups. Pan-macrophages were identified as CD68-positive (**A–C, E–G**), and M1 or M2 cells were recognized as positive expression of the cell surface marker CCR7 (**I–K, M–O**) or CD206 (**Q–S, U–W**). Level of the pan-macrophage (M) marker CD68, the M1 marker CCR7, and the M2 marker CD206 were statistically analyzed (**D, H, L, P, T, X**). All data are given as the mean ± SEM (*n* = 10). *p < 0.05, **p < 0.01

IHC analysis showed that from 1 to 2 weeks, T-bet^+^ Th1 and ROR gamma T^+^ Th17 counts in the EPL/PCL/HA group were decreased 50–60% than those in the PCL and PCL/HA scaffolds (Fig. 2A–H, Q–X). The Th1 count in the PCL/HA group was higher 1 time than that of the PCL and EPL/PCL/HA groups at both 1 and 2 weeks (Fig. 2A–H). Although there was no significant difference in GATA3^+^ Th2 count between the three groups at 1 week, the count in EPL/PCL/HA group was upregulated about 25% than PCL/HA at 2 weeks (Fig. 2I–P). In addition, our results showed that the number of CD68^+^ pan-macrophage in PCL/HA and EPL/PCL/HA groups were only about 30% of PCL group at 1 week (Fig. 3A–D). At 2 weeks, the number of pan-macrophage in EPL/PCL/HA group was 55% of PCL/HA and PCL groups (Fig. 3E–H). The CCR7^+^ M1 count in EPL/PCL/HA group was lowest in all the three groups at 1 week, which was only 50% of other groups (Fig. 3I–L), but there was no significant difference in all the groups at 2 weeks (Fig. 3M–P). The CD206^+^ M2 count in PCL/HA and EPL/PCL/HA groups was only 70% of PCL group at 1 weeks, while was significantly increased to two times of PCL at 2 weeks, no difference was found between PCL/HA and EPL/PCL/HA group (Fig. 3Q–X). However, the EPL/PCL/HA group pan-macrophage count was the lowest of the three groups, suggesting that EPL/PCL/HA may promote pan-macrophage polarization into type M2.

### Toluidine blue dye and fluorescence analysis showed EPL/PCL/HA promote bone defect repair in rabbit calvarial bone defect

**Fig. 4:**
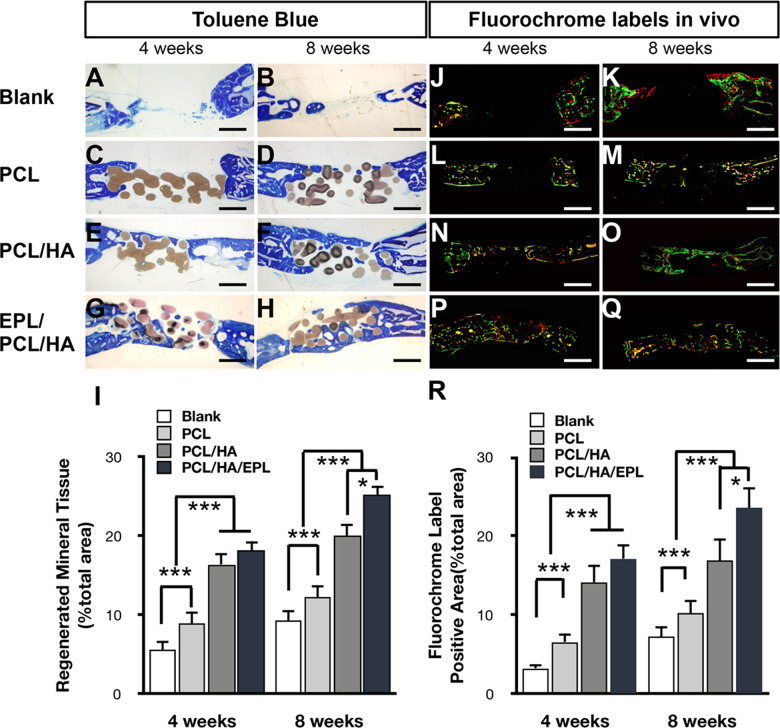
Histological observations made using fluorescence imaging and toluidine blue staining at weeks 4 and 8, respectively. **A–H** Histological evaluation of new bone formation in the blank, and PCL, PCL/HA, and EPL/PCL/HA scaffold groups. Histomorphometric analysis of the newly formed bone. **I** Regenerated mineral tissue (% total area) is reflected by the ratio of new tissue area to the total scaffold area (*n* = 10, *p < 0.05). **J–Q** Fluorescent labeling of calcein and alizarin red. The newly formed bone was labeled with calcein (diffuse green) and alizarin red (thin red lines). Merged images of the two fluorochromes. **R** Percentage of calcein and alizarin red staining for each group assessed at weeks 4 and 8 after implantation as revealed by histomorphometric analysis. *p < 0.05, ***p < 0.001

Toluidine blue staining of new mineral tissue on the edge of the bone defect and the bone tissue surface were stained blue, and a small amount of staining appeared in the middle region of the bone defect. Our data showed that at 4 and 8 weeks after implantation in rabbit calvarial bone defect, significantly more new mineral tissue formation occurred in the EPL/PCL/HA and PCL/HA groups than in the blank and PCL groups (Fig. 4A–I). Furthermore, the percentage of newly mineralized tissue in the EPL/PCL/HA group was about 1.3 times of PCL/HA, 2 times of PCL, and 3 times of blank groups at weeks 8 (Fig. 4I).

Fluorescence microscopy analysis showed that the host bone was melanocratic and unlabeled, whereas calcein and alizarin red labeled the newly formed bone with a diffuse green pattern and thin red lines, respectively. At 4 weeks, our results showed no obvious new mineral tissue formation in the blank group, while a small amount of irregular and asymmetric mineral tissue had formed at the defect edge in the PCL, PCL/HA, and EPL/PCL/HA groups, and the shape of the scaffolds was well-maintained (Fig. 4J, L, N, P). At 8 weeks, the amount of new mineral tissue formation in all the three groups increased significantly. The surface of the scaffold material was gradually replaced by new mineral tissue (Fig. 4K, M, O, Q). Quantity analyses showed that most new mineral tissue formed in the EPL/PCL/HA group, followed by the PCL/HA and PCL groups. The lowest new mineral tissue formation occurred in the blank (control) group (Fig. 4R).

### H&E and Masson’s trichrome staining

**Fig. 5:**
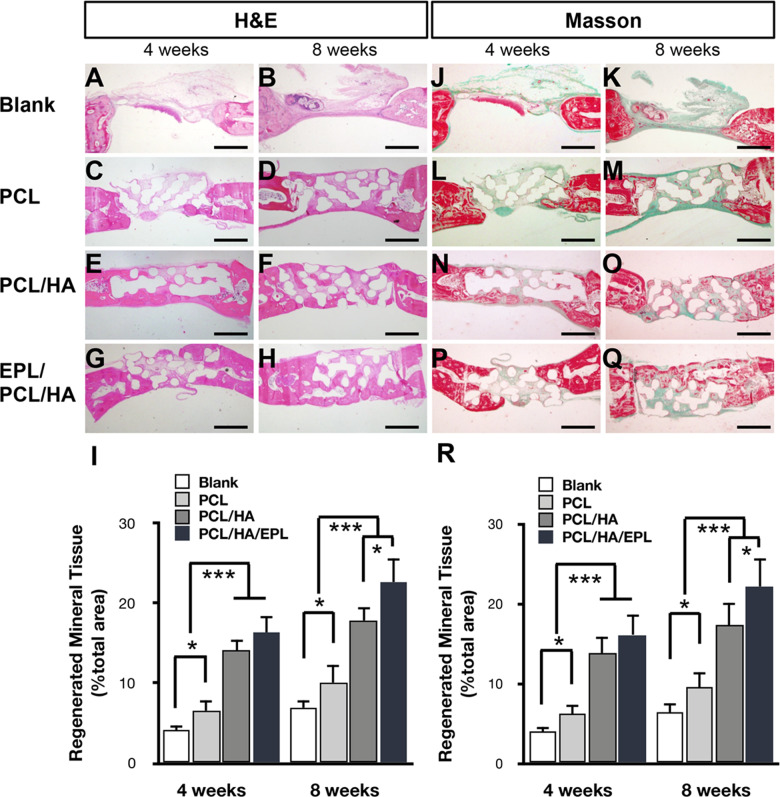
Histological features of the four groups stained with hematoxylin & eosin and Masson’s trichrome staining. **A–Q** Histological findings at weeks 4 and 8. Histological evaluation of new bone formation in the blank, and PCL, PCL/HA, and EPL/PCL/HA scaffold groups. Histomorphometric analysis of the newly formed bone. Regenerated mineral tissue (% total area) shown is reflected by the ratio of new tissue area to the total scaffold area. **I, R** (*n* = 10, *p < 0.05, ***p < 0.001)

H&E and Masson’s trichrome staining were used to evaluate the regenerated tissue in each group. The results showed that the new mineral tissue was bone-like tissue. At 4 weeks, little newly formed bone migrated from the defect periphery in the blank and PCL groups. More newly formed bone was detected in the PCL/HA and EPL/PCL/HA groups, the EPL/PCL/HA group showed the highest amount of new bone formation (Fig. 5). When the implantation time was extended to 8 weeks, more newly formed bone was found in the PCL, PCL/HA, and EPL/PCL/HA groups compared to implantations at week 4, while only a small amount of new mineral tissue formed in the blank group (Fig. 5). The highest amount of newly formed mineral tissue was observed in the EPL/PCL/HA group, which was 1.3 times of PCL/HA, 2 times of PCL, and 3 times of blank, demonstrating the strong bone defect repair capacity of EPL/PCL/HA scaffolds (Fig. 5I, R).

## Discussion

The present study showed that EPL/PCL/HA scaffold suppressed pro-inflammatory Th1, Th17, and M1 levels and promoted anti-inflammatory Th2 infiltration into regenerated areas in vivo. It also suppressed pan-macrophage levels and may promote pan-macrophage polarization into anti-inflammatory M2 cells. In addition, our results showed that EPL/PCL/HA enhanced tissue growth and bone defect repair capacity in a rabbit model.

Despite significant differences in long-term results, all implants elicit similar histological cellular responses from 1 week to 1 month after implantation, which is characterized by an early infiltration of neutrophils followed by a dense infiltration of monocytes [29, 30]. Innate immune system is involved in the first phase of immune response, which can be activated in hours and last for days [31]. Macrophages are differentiated from monocytes about 1 week, are pivotal for the ossification process [32]. It has pro-inflammatory, wound healing, anti-inflammatory, and tissue-regenerating functions when they become polarized and differentiate into pro-inflammatory M1 macrophages and anti-inflammatory M2 macrophages [33–35]. In the early stages of external stimulation (wound healing and infection), macrophages first appear as pro-inflammatory M1 macrophages, secreting inflammatory mediators (TNF-α, IL-1, and nitric oxide) and activating antibacterial defense mechanisms, including the oxidation process, to kill microorganisms that invade the body. In the later stage of infection, the body regulates and weakens the inflammatory response to avoid damaging the body [36]. The regulation mechanism switches the macrophage polarization types, shifting them from M1 to M2 macrophages, or induces apoptosis. The generated M2 macrophages slow down the inflammatory response and promote tissue repair and wound healing [37]. Disruption of the shift from M1 to M2 can cause tissue damage. The adaptive immune response system is activated within a week. The infiltration of immune cells (Th1, Th2, Th17) into a transplant area is inevitable, and certainly, immune cell infiltration is necessary for tissue regeneration and wound healing [36, 38]. However, substantial pro-inflammatory cell infiltration caused by infection or degradation impairs tissue regeneration and can lead to acute graft-versus-host disease (aGVHD) [39]. GVHD is caused by cytotoxicity mediated by donor T-cell recognition recipients with different histocompatibility antigens [40]. Cytokines produced and released by Th1 cells (Th1 cytokines, IFN-γ, and TNF-α) are involved in, and promote the occurrence of, aGVHD. A recent study showed that IL-17 produced by Th17 cells is involved in aGVHD occurrence and development [41]. While Th2 cytokines inhibit IFN-γ secretion and reduce aGVHD occurrence, they can also reduce the excessive secretion of inflammatory cytokines in the host tissues and organs, thereby balancing the production of Th1/Th2/Th17 cytokines, alleviating aGVHD, and protecting the host tissues and organs [42].

Consistent with this, our study showed high levels of pro-inflammatory M1 macrophages (Th1 and Th17) at 4 weeks after implantation, while at 8 weeks, the majority of lymphoid cells were reversed to anti-inflammatory M2 and Th2 types in all groups. Our results showed that PCL, PCL/HA, and EPL/PCL/HA scaffolding have good biocompatibility and promote a normal healing process. The EPL/PCL/HA scaffold decreased the rate of Th1, Th17, and pan-macrophages at both 4 and 8 weeks, and increased the rate of M2 macrophages. This earlier reversal of pro-inflammatory to anti-inflammatory cells promoted by the EPL/PCL/HA scaffold explains its superior bone defect repair capacity. This may be because the amine and hydroxyl groups contained in EPL itself can mimic the extracellular matrix (adhesin, glycosaminoglycan, fibrin, etc.) and microenvironmental changes inside the 3D grid scaffold caused the aggregation of anti-inflammatory cells into the scaffold, however, the exact mechanism needs further exploration. Our data also found that PCL/HA had a similar but weaker inflammation regulation function to EPL/PCL/HA, except it induced a higher rate of Th1 at 4 and 8 weeks in vivo; it is possible that the HA particle we used triggered Th1 infiltration as different HA nanostructures present different Th1 responses [43].

PCL, as a semi-crystalline artificial polymer with good mechanical properties, low melting point, biodegradability, and easy processing and shaping, can be used safely in the human body and has been used in a number of clinical applications. Combined with HA, PCL/HA has been confirmed has good biological performance and widely used in bone reconstruction. Many studies also optimize the hybrid PCL scaffolds, such as using strontium (Sr) containing HA or silicate (Si) containing HA to build hybrid PCL/SrHA or Si-HA, which have increased wettability, degradation rate, cell adhesion, and proliferation compared with hybrid PCL/HA scaffolds [44–46]. Although the hybrid PCL/SrHA or Si-HA scaffolds may be more effective, the PCL/HA scaffolds were used for this study to show the immunoregulation of EPL. The EPL coated with hybrid PCL/SrHA or Si-HA scaffolds would be investigated in the future.

EPL is a biodegradable polymer that can be degraded into small molecule amino acids by hydrolysis or enzymatic reactions and absorbed by the body. Its ability to promote tissue healing and inhibit bacterial growth make it a suitable ingredient of scaffold for craniomaxillofacial defect repair as the craniomaxillofacial is an area with high bacterial density. EPL/PCL/HA scaffolding promoted tissue growth inside the defects, thus accelerating tissue growth [47]. There are several possible mechanisms that may explain this property: first, our previous study has showed that the EPL/PCL/HA scaffold surface has enhanced hydrophilicity [27], which was due to HA offering the free –OH group and the amidogens of EPL bound with proteoglycans on the cells’ surface [48–50], making it easy to maintain the scaffold structure and improving cell adhesion. Second, EPL can directly interact with cell lipid bilayers, preventing cells from agglomerating into spheres through electrostatic attraction and other functions. This causes cells to adhere to the material and spread into a single layer, which significantly improves cell adhesion efficiency under 3D conditions [51]. Last, EPL degrades into lysine, or amine, amide, hydroxyl, or carboxyl groups that can promote cell adhesion and growth.

## Conclusion

In this study, 3D printed PCL/HA scaffold coated by 5-mg/ml ε-poly-l-lysine (EPL) have been successfully build as a new EPL/PCL/HA composite scaffold. When implanted into the rabbit muscle, it showed that the EPL/PCL/HA promotes tissue regeneration in the muscle transplant model and decreased pro-inflammatory Th1, Th17, and type I macrophage infiltration, and increased anti-inflammatory Th2 infiltration into the regenerated area, when compared to PCL and PCL/HA. In addition, EPL/PCL/HA showed an enhanced bone repair capacity compared to PCL and PCL/HA when transplanted into rabbit calvarial bone defects at both 4 and 8 weeks. Thus, our results suggest that EPL is a promising agent for tissue engineering scaffold modulation.

## Supplementary information

Supplementary Figure 2

Supplemental Figure 1
