# Supplementary figures and images for: The immunogenic reaction and bone defect repair function of ε-poly-L-lysine (EPL)-coated nanoscale PCL/HA scaffold in rabbit calvarial bone defect
Source: J Mater Sci Mater Med. 2021 Jun 7;32(6):63. doi: 10.1007/s10856-021-06533-7 (PMC8184523; doi:10.1007/s10856-021-06533-7)

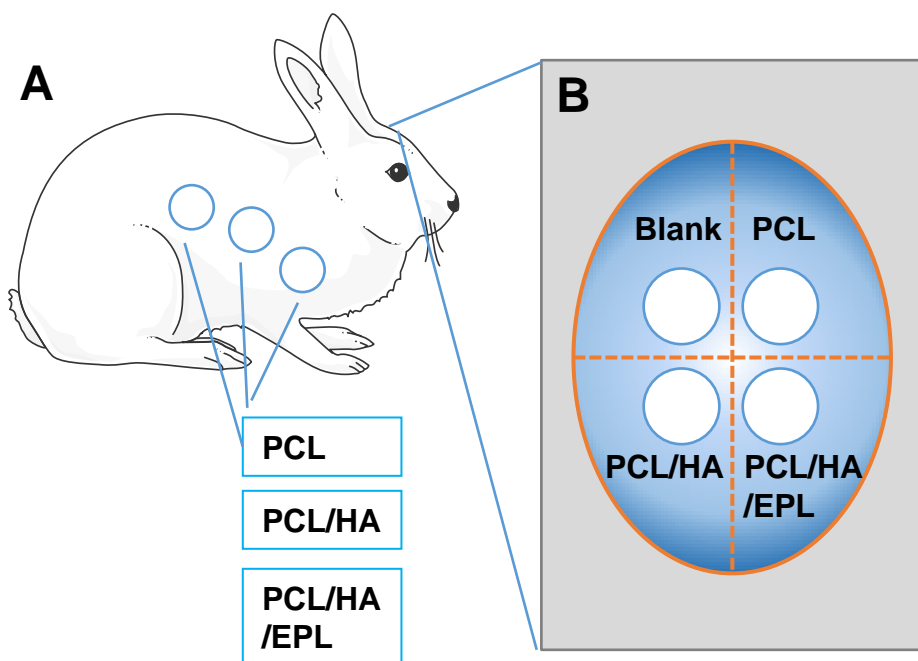

**4 Weeks**

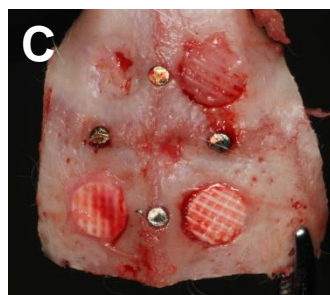

**8 Weeks**

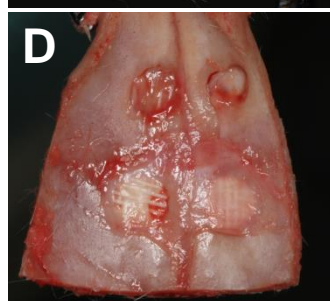

Supplement: Supplementary file 1 — Supplementary Figure 2 [file 10856_2021_6533_MOESM1_ESM.pdf]
