# Supplementary material for: The immunogenic reaction and bone defect repair function of ε-poly-L-lysine (EPL)-coated nanoscale PCL/HA scaffold in rabbit calvarial bone defect
Source: J Mater Sci Mater Med. 2021 Jun 7;32(6):63. doi: 10.1007/s10856-021-06533-7 (PMC8184523; doi:10.1007/s10856-021-06533-7)
Supplement: Supplementary file 2 — Supplemental Figure 1 [file 10856_2021_6533_MOESM2_ESM.docx]

Supplemental Figure 1. The implantation sites in the vivo studies

A: The sites of the PCL, PCL/HA, and EPL/PCL/HA scaffolds implanted into the rabbit muscle. B: The sites of scaffold implantation in rabbit skull bone defects of critical size. C,D: the gross of 4 weeks and 8 weeks.
